# Soft Smart Garments for Lower Limb Joint Position Analysis

**DOI:** 10.3390/s17102314

**Published:** 2017-10-12

**Authors:** Massimo Totaro, Tommaso Poliero, Alessio Mondini, Chiara Lucarotti, Giovanni Cairoli, Jesùs Ortiz, Lucia Beccai

**Affiliations:** 1 Center for Micro-BioRobotics, Istituto Italiano di Tecnologia, Viale Rinaldo Piaggio, 34, 56025 Pontedera, Italy; alessio.mondini@iit.it (A.M.); chiara.lucarotti@iit.it (C.L.); 2 Department of Advanced Robotics, Istituto Italiano di Tecnologia, Via Morego, 30, 16163 Genova, Italy; tommaso.poliero@iit.it (T.P.); giovanni.cairoli@iit.it (G.C.); jesus.ortiz@iit.it (J.O.)

**Keywords:** wearable system, strain sensor, bending, soft tactile sensor, textile, capacitive sensor, exoskeleton, human motion monitoring

## Abstract

Revealing human movement requires lightweight, flexible systems capable of detecting mechanical parameters (like strain and pressure) while being worn comfortably by the user, and not interfering with his/her activity. In this work we address such multifaceted challenge with the development of smart garments for lower limb motion detection, like a textile kneepad and anklet in which soft sensors and readout electronics are embedded for retrieving movement of the specific joint. Stretchable capacitive sensors with a three-electrode configuration are built combining conductive textiles and elastomeric layers, and distributed around knee and ankle. Results show an excellent behavior in the ~30% strain range, hence the correlation between sensors’ responses and the optically tracked Euler angles is allowed for basic lower limb movements. Bending during knee flexion/extension is detected, and it is discriminated from any external contact by implementing in real time a low computational algorithm. The smart anklet is designed to address joint motion detection in and off the sagittal plane. Ankle dorsi/plantar flexion, adduction/abduction, and rotation are retrieved. Both knee and ankle smart garments show a high accuracy in movement detection, with a RMSE less than 4° in the worst case.

## 1. Introduction

With the emergence of soft materials and related technologies, there was an increase in research efforts aimed at developing soft wearable sensing systems [1,2] that can intimately interface to the wearers. A critical requirement for sensors in soft wearable systems is that they must conform to the body’s geometry and to soft tissues without impeding the natural and nonlinear motions of the body itself. Soft wearable sensors, attached to the human body in the form of clothes or accessories, can aid health diagnosis and detect emergency situations by monitoring vital parameters (e.g., body temperature, blood pulse, and electromyogram) [3,4,5,6,7]. In addition to measuring physical parameters on the human body [8,9], they can enable monitoring external contact forces and body motion in real time, and transfer data to electronic devices for different useful applications, including healthcare (biomechanics, rehabilitation, etc.) and entertainment (sports, gaming, etc.). Designing wearable sensors is a very challenging task since many highly constraining, and often conflicting, requirements have to be considered. For instance, the mechanical properties of the constituent materials are a key issue: they have to be flexible and stretchable enough to allow the complete adaptation to the body and to follow its reversible deformations. In addition, the weight and size factor of the system need to be accounted for, so that the system does not hinder any of the user’s normal movements or actions. Furthermore, robustness and repeatability issues need to be accounted for both the output signals, and the mechanical characteristics of the specific combination of employed materials.

Despite these challenges, a continuous evolution of new wearable sensing devices has taken place throughout the years for diagnostics as well as monitoring. Their capabilities include physiological and biochemical sensing, as well as motion sensing and gait analysis [10,11,12]. For example, physiological monitoring [13,14,15] could help in both diagnosis and ongoing treatment of a vast number of individuals with neurological, cardiovascular and pulmonary diseases such as seizures, hypertension, dysthymias, and asthma. Home-based motion sensing might assist in falls prevention and help maximize an individual's independence and community participation [16].

In the case of human movement monitoring, in recent years, several solutions were proposed. For instance, inertial measurement units (IMUs) [17,18,19] that are based on a combination of accelerometers and gyroscopes integrated in one miniaturized system, have shown a remarkable development driven by the advancements in both electronic miniaturization and signal processing. While their cost is constantly diminishing and performances are improving, they are unfortunately made of rigid components that can affect long-term wearability and widespread diffusion. In this view, recently some new IMU units, including data storage and communication components, have been embedded in flexible packages that can adhere directly to the skin [20,21,22].

Considering the transduction principle used in the sensing systems, mainly resistive [23,24] and capacitive [25,26] approaches were exploited in the past. In both cases, strain causes a mechanical deformation in the sensor, which is transduced in a resistive or capacitive variation. While resistive sensors are cheaper and simpler to fabricate, they present some drawbacks for long term applications. In particular, they have a large hysteresis, and high sensitivity to temperature and humidity variations [1,27,28]. These parameters should be considered properly in wearable systems, since they can vary largely due to the environment and the contact with the body (i.e., sweat). On the other hand, capacitive sensors do not present the abovementioned disadvantages while having a higher sensitivity, large dynamic range, long-term drift stability, lower power consumption as compared to piezoresistive/resistive devices. However, they need a more complex read-out electronics and strategies to minimize proximity effects and parasitic elements. In particular, wiring and connections must be shielded, without increasing encumbrance or affecting flexibility and wearability of the overall system, and these aspects are not trivial.

Indeed, the main requirement is having an unobtrusive system, but at the same time reliable and durable. In this view, a possible approach is detecting the body surface strain due to the bending of joints by means of a fully conformable sensorized garment. The latter must extend and release following the skin without sliding above it, but at the same time it must be comfortable to wear in order to not affect human movements. In this route, it is reasonable to take all the advantages of modern textiles and use those also to build the sensing parts integrated in the clothing. In recent years, textile-based sensors and electrodes had a boost due to several novel materials such as graphene [29,30], conductive polymers [31,32], nanoparticles [33,34], carbon nanotubes (CNTs) [35,36] used to make conductive patterns in the garment.

Several commercial conductive textiles [37,38,39], with different mechanical and electrical properties, have also appeared on the market. They can be integrated in soft exoskeletons [40,41] for motion monitoring and rehabilitation. However, while showing relevant advancements, they still have some limitations. In particular, in the case of lower limbs, they can sense movements mainly in the sagittal plane [40] (i.e., the knee bending and the ankle flexion/extension). This limits the analysis and the assistance of several mobility impairments that involve more complex movements (off the sagittal axis). Moreover, in the case of embedding soft capacitive sensors [25], a simple two-electrode structure is not immune to proximity effects and electromagnetic interferences, and, in addition, it is intrinsically sensitive to applied pressure. Then, proper strategies should be adopted to overcome these factors.

In this work, we present sensorized wearable modules, for monitoring the movements of both knee and ankle joints, developed in the framework of H2020-EU XoSoft project [42,43,44] that aims to build new exoskeletons to assist people with mobility impairments. Capacitive sensing elements, made of a combination of conductive textile and elastic polymers, are integrated in commercial knee and ankle braces, together with a removable wireless readout electronic system. The sensors, due to their structure, are shielded from proximity effects and electromagnetic noise. The textile approach is crucial since it allows one to achieve monitoring of individuals at low cost and to easily customize the sensor configuration according to the needs of each subject.

Both knee and ankle modules are characterized employing an optical tracking system. Combining in a proper way sensor outputs different information are retrieved: for the knee, the bending angle and the occurrence of possible external contacts; for the ankle, the three-dimensional motion monitoring is addressed, by correlating the sensor outputs with the three Euler angles in the case of basic movements (i.e., dorsi/plantar flexion, adduction/abduction, and rotation).

## 2. Materials and Methods

### 2.1. Capacitive Textile-Based Strain Sensor

**Figure 1:**
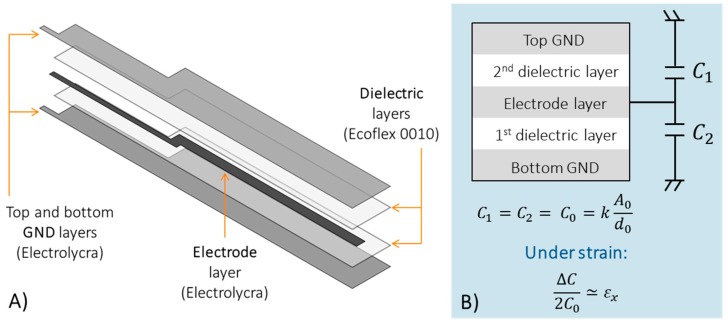
Technological (**A**) and electrical (**B**) scheme of the three-electrode soft sensor configuration.

The capacitive strain sensor consists of a combination of conductive and dielectric layers, and in its design shielding issues had to be addressed. Hence, a simple capacitor structure cannot be used [45], instead a three-electrode configuration was implemented (see Figure 1). In particular, the bottom and top electrodes of the capacitor were connected to ground, while the central electrode provides the sensing signal to the conditioning electronics. In this way, parasitic capacitances and proximity effects were drastically reduced. In addition, both bottom and top ground electrodes were designed larger than the other layers (central electrode and dielectric layers) in order to completely embed the sensing volume, and to shield it. Parasitic effects must be avoided also in the electrical connections from the sensor to the electronic circuitry, thus, shielded connections were implemented by using the same three-electrode configuration up to the electronic board. Therefore, shielded sensor and connection result as a whole system integrated in the textile.

From an electrical point of view, the nominal capacitance of the soft strain sensor $C_{0\_tot}$, in the three-electrode configuration, can be considered as the parallel between $C_{0_{1}}$ and $C_{0_{2}}$ (i.e., the capacitance between the electrode and the bottom and top ground layers, respectively). In particular, $C_{0_{1}}$ and $C_{0_{2}}$ can be considered as the capacitances of two parallel electrodes plates, as follows:

(1) $$C_{0_{1}}=\frac{k_{0}k_{r_{1}}A_{1}}{d_{0_{1}}}$$

(2) $$C_{0_{2}}=\frac{k_{0}k_{r_{2}}A_{2}}{d_{0_{2}}}$$

where $k_{0}$ is the dielectric constant of the free space (~8.85 pF/m), $k_{r_{1}}$ and $k_{r_{2}}$ are the relative permittivity for the first and the second dielectric material, $A_{1}$ and $A_{2}$ are the sensing area between the electrode and the top and bottom ground layers, $d_{0_{1}}$ and $d_{0_{2}}$ are the dielectric thicknesses. Therefore, $C_{0\_tot}$, which is the parallel between $C_{0_{1}}$ and $C_{0_{2}}$, is given by Equation (3):

(3) $$C_{0\_tot}=C_{0_{1}}+C_{0_{2}}=k_{0}\left(\frac{k_{r_{1}\;}A_{1}}{d_{0_{1}}}+\frac{k_{r_{2}}A_{2}}{d_{0_{2}}}\right)$$

Considering the use of the same material for both dielectric layers (i.e., $k_{r_{1}}=k_{r_{2}}=k_{r}$), and with the same thicknesses (i.e., $d_{0_{1}}=d_{0_{2}}=d_{0}$), and the same sensing area between the electrode and the top and bottom ground layers (i.e., $A_{1}=A_{2}=A_{0}=l_{0}w_{0}$), the nominal capacitance $C_{0\_tot}$ results:

(4) $$C_{0\_tot}=2k_{0}k_{r}\frac{A_{0}}{d_{0}}=2C_{0}$$

When a longitudinal strain $\varepsilon_{x}=\left(l-l_{o}\right)/l_{0}$ is applied to the sensor, both the dielectric thicknesses and the sensing area vary, resulting in a capacitance variation ΔC with respect to the nominal value $C_{0\_tot}$, as explained in the following Equation (5):

(5) $$\Delta C=C-C_{0_{tot}}=\;2k_{0}k_{r}\left[\frac{\left(1+\varepsilon_{x}\right)l_{0}\left(1-\nu_{el}\varepsilon_{x}\right)w_{o}}{\left(1-v_{d}\varepsilon_{x}\right)d_{0}\;}\right]-2k_{0}k_{r}\frac{A_{0}}{d_{0}}≃2C_{0}\varepsilon_{x}$$

Assuming $\nu_{el}=\nu_{d}$ (i.e., the same Poisson ratio for both electrode and dielectric layer).

### 2.2. Knee Module

The analysis of the knee bending movement reveals that the largest strain occurs in the sagittal axis direction. According to this, the strain sensors for the knee brace were designed in rectangular shape, with the longest side parallel to the sagittal axis. In this way, the deformation of the sensors, and therefore the output signals, is maximized. More specifically, the sensorized knee brace integrates three sensors. One sensor is positioned at the knee centre in correspondence of the kneecap (named C2), while the two other sensors are placed at kneecap’s sides (named C1 and C3). Each sensor has a sensing area of 162 mm^2^. The capacitive elements are sensitive also to pressure solicitations; therefore, the integration of three sensors allows the simultaneous discrimination between strain (due to the bending of the knee) and pressure (due to accidental contact with the surrounding). The sensors are connected by shielded connections to an electronic board that will be described in Section 2.5.

### 2.3. Ankle Module

**Figure 2:**
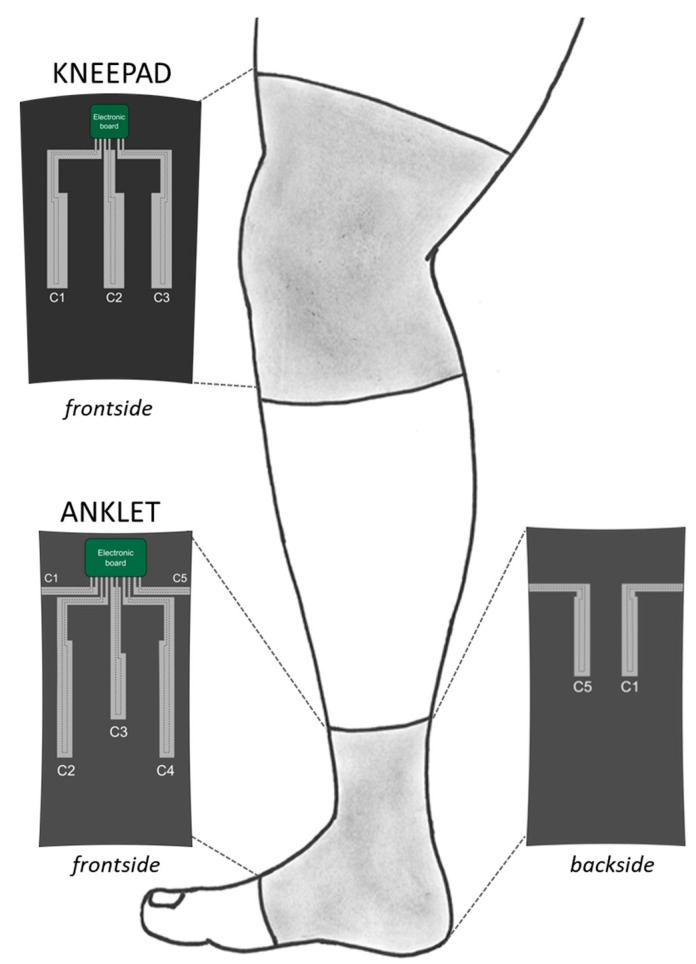
Schematic showing a sketch of a human lower limb with the design of knee and ankle modules integrating a sensing part with a specific layout, and an electronic readout system.

For the general purpose monitoring of ankle angles relative to dorsi/plantar flexion, abduction/adduction, and rotation, five strain sensors were integrated in the commercial ankle brace (Figure 2). Three sensors were integrated on the front side of the ankle brace: one central (named C3 with a sensing area of 93 mm^2^) and two lateral sensors (named C2 and C4, each having an area of 180 mm^2^). The other two sensors were positioned on the back side of the brace (i.e., C1 and C5 with a sensing area of 120 mm^2^ for each). By combining all sensor outputs with a proper algorithm, the monitoring of the desired movements is possible, as shown below.

### 2.4. Smart Garment Fabrication

Conductive textiles and non-conductive silicone elastomers were selected as constituent materials because of excellent mechanical properties in terms of stretchability, flexibility and compliance. They can be combined adhering to each other in the multilayer structure reported in Section 2.5, while at the same time they allow proper conformability to the garment in which they are integrated. This way the resulting soft capacitor responds to any reversible change in the deformation caused by the movement of the joints, and it can be smoothly stimulated even by very subtle wearer movements.

As shown in Figure 1, the conductive parts of the sensor were built with Electrolycra (Mindsets Online, Middlesex University, London, UK) stretchable conductive textile, which is made by a combination of nylon and elastic fibres, and plated in silver. The choice of such stretchable conductive fabric for the electrodes is fundamental since it is mechanically very robust. On the other hand, Ecoflex^®^ silicone elastomer (Ecoflex 00-10, Smooth-On Inc., Macungie, PA, USA) with a dielectric constant $k_{r}$ of 2.5, was chosen for the dielectric layers of the shielded capacitor. 

The design of the sensor is the result of a trade-off between two major aspects: (i) the overall thickness should be minimized to improve wearability; and (ii) the thickness *d*_0_ of the dielectric layers must allow a suitable output signal due to a capacitance variation. The latter is the result of a trade-off between the obtainable displacement of the electrodes affected by the soft dielectric deformability, and the value effectively needed for a proper *C*_0_.

**Figure 3:**
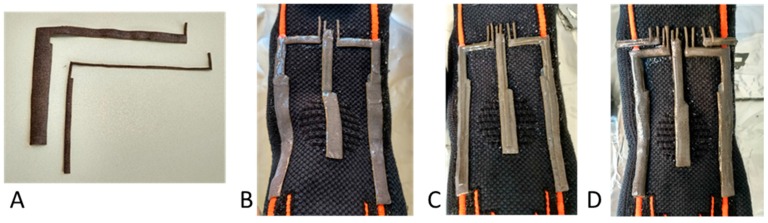
Fabrication process: (**A**) Laser cutting of ground and sensing electrodes. Deposition of: (**B**) Electrolycra bottom ground electrodes and first dielectric layers; (**C**) Central electrodes and second dielectric layers; (**D**) Top ground electrodes and external passivating layers.

The capacitive strain sensors were assembled *layer-by-layer* directly onto the knee and ankle braces to achieve an integrated solution. The fabrication steps are summarized in the following, and different steps are shown in Figure 3:

1. At first, Electrolycra electrodes (having 600 μm thickness) were cut at the desired shape by CO_2_ laser cutter (VLS 3.50, Universal Laser System, Inc., Scottsdale, AZ, USA) with a resolution of 0.5 mm. It should be noted that the patterns include textile at the extremities for the wiring, as explained in step (5);
2. As anticipated, the different layers were deposited on the fabric of commercially available kneepad (made of 80% polyester and 20% elastodiene) and anklet (consisting of 57% polyamide, 26% elastodiene, 13% polyester, and 4% elastane); At the same time, Ecoflex^®^ was spin-coated from a 1:1 (weight/weight) solution of Ecoflex^®^—Part A and—Part B on a silicon wafer, at 1200 rpm for 60 s, and then cured for 4 h at room temperature to obtain dielectric films with desired thickness of 130 µm;
3. The conductive (bottom ground, central electrode and top ground) and dielectric (between each couple of electrodes) layers of the sensors were glued to each other by dispensing a small quantity of uncured Ecoflex^®^;
4. A small quantity of uncured Ecoflex^®^ was deposited to encapsulate and insulate the sensors, and the curing of the whole device was performed at room temperature for 4 h;
5. Finally, textile wiring, patterned at each electrode, is used for making the connection between the sensors and the read-out circuitry. In this way, the length of connections is minimized, in order to keep parasitic capacitances as low as possible, and the wiring results shielded and embedded in the garment, like the sensors.

**Figure 4:**
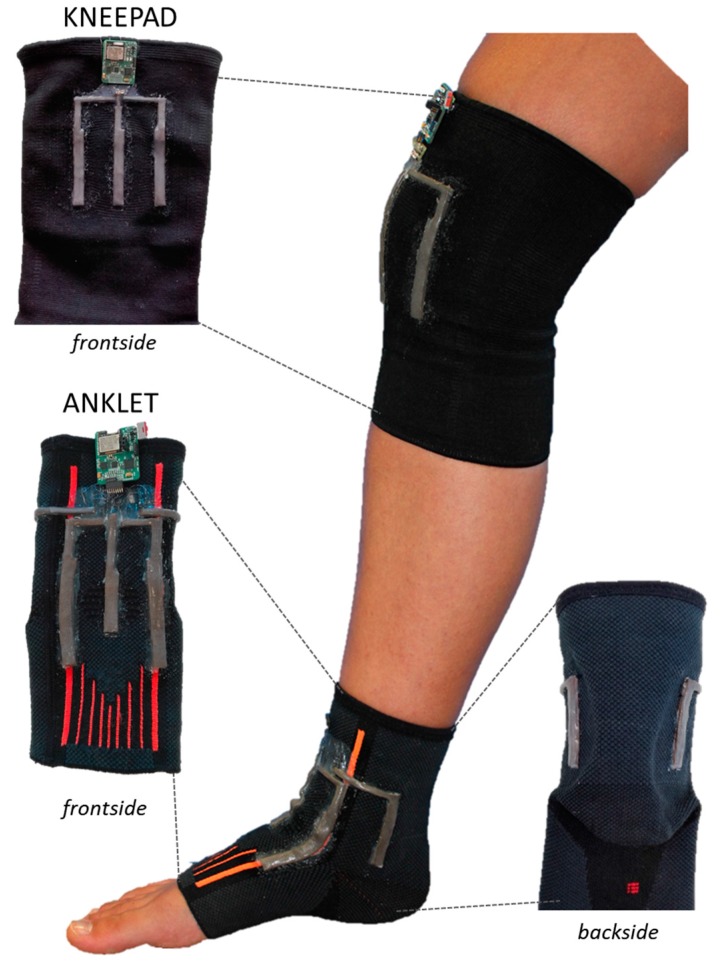
Prototypes of knee and ankle modules each integrating several sensors connected to one read out electronic system that communicates with an external board by a RF module.

In this work, the thickness of Ecoflex^®^ layer deposited in step (3), although not being precisely controlled, was qualitatively kept to a minimum thickness. Indeed, it must be considered that each prototype undergoes a calibration procedure when this variability can be compensated. Both modules, worn by a user and equipped with the read-out electronic system, are shown in Figure 4.

### 2.5. Read-Out Electronic System

Together with the sensors, customized capacitance readout electronic system was built. It consists of a 16-bit multichannel capacitance-to-digital converter (AD7147, Analog Device Inc., Nordwood, MA, USA) with 1 fF resolution, a 32-bit PIC microcontroller (PIC32MX150F128B, Microchip Technology Inc., Chandler, AZ, USA), and a RF module (RFD21733, RF Digital Corp, Hermosa Beach, CA, USA) to transmit data to the control PC without additional wiring. The final dimension of the board is 2.7 cm × 3.4 cm.

The electronic system permits the acquisition of up to 8 capacitive signals, and a transmission rate of 10 Hz. Data are received by a twin RF module in a second electronic board connected via USB to a PC for data collection and visualization in real time by means of a user interface developed in Visual Basic .NET (Microsoft, Redmond, WA, USA).

## 3. Results

### 3.1. Single Strain Sensor

The characterization of the sensorized braces was addressed in two different phases, i.e., the characterization of the individual electrotextile-based strain sensor and the characterization of the sensorized knee and ankle braces.

**Figure 5:**
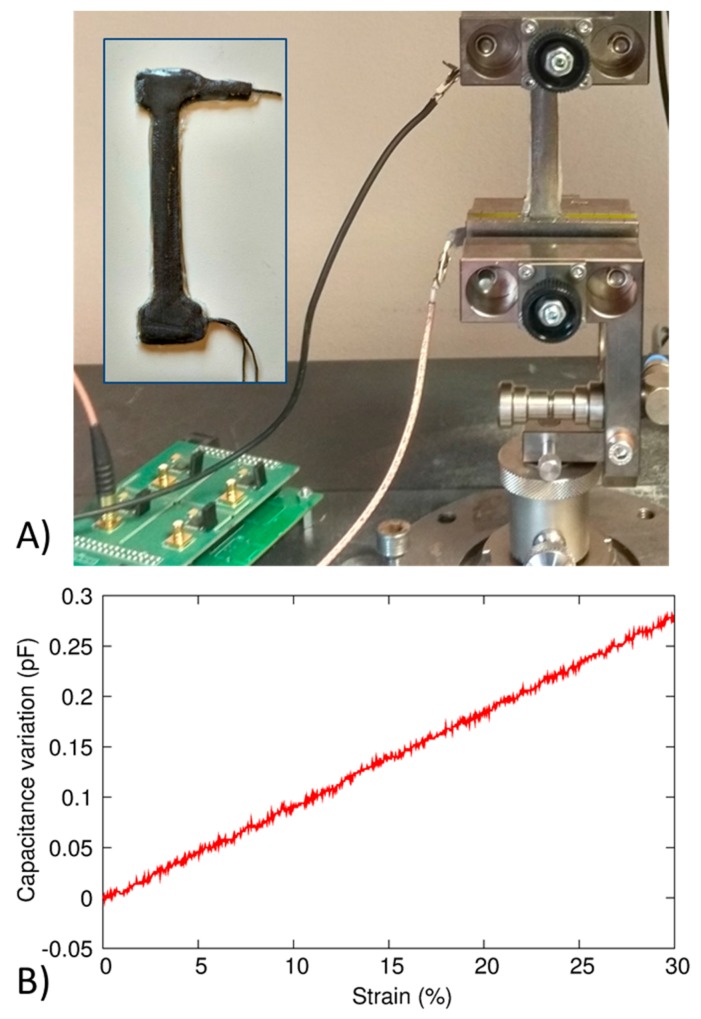
(**A**) Experimental setup for the characterization of the single capacitive strain sensor; (**B**) Capacitive variation vs. strain output characteristic.

Firstly, the individual electrotextile-based strain sensor was tested to evaluate its behaviour in terms of capacitance variation versus applied strain. To this aim, a sensor with dumbbell shape (90 mm of total length), shown in the inset of Figure 5A, and three-electrode configuration was developed by using the same constituent materials previously described. The sample was characterized by means of tensile tests (10 Hz acquisition frequency, 10 mm/min velocity) with a universal testing machine (Zwick/Roell Z005, Ulm, Germany) equipped with a load cell detecting forces up to 50 N (see Figure 5A). The sensor was clamped at both extremities, and the area subjected to strain was 4.5 mm × 68 mm. Strain data were acquired by the testing machine, while capacitance measurements were done by means of a custom electronics to which the sensor was connected by shielded cables. The characteristic is shown in Figure 5B. The sensor was characterized up to 30% of strain, higher than the maximum elongation occurring in typical lower limb movements, as shown in next sessions. A linear behaviour can be observed in the whole range.

### 3.2. Characterization System and Reconstruction Algorithm

**Figure 6:**
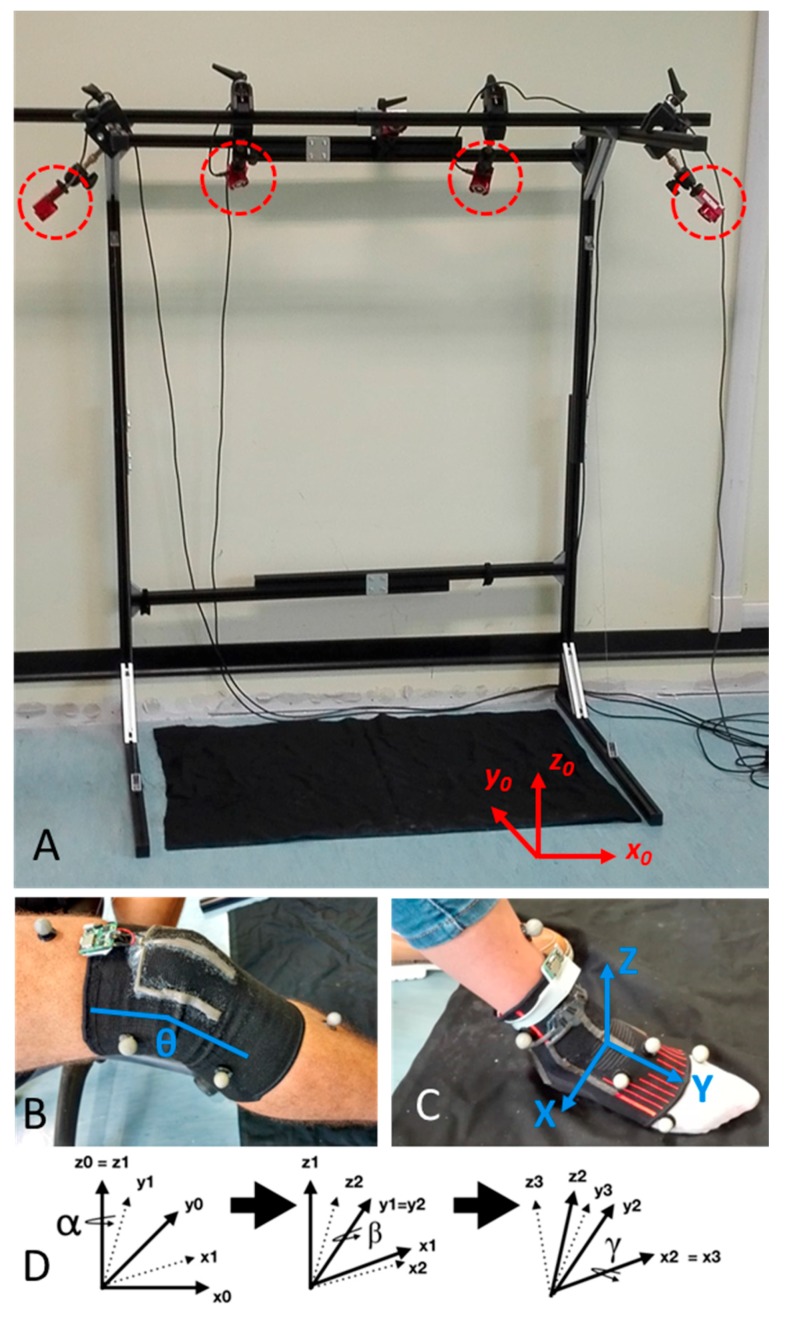
(**A**) Optitrack system used for experimental characterization. In red circles the four cameras of the system. The *x*_0_-*y*_0_-*z*_0_ fixed reference system is displayed in red. (**B**) Knee module worn during experiments. Optical markers, attached to the body, are well visible. The knee bending angle *θ* is labeled in blue. (**C**) Ankle module worn during experiments, with optical markers attached to the body. The moving reference system *x*-*y*-*z* is labeled in blue. (**D**) Euler angles used in the reconstruction algorithms for the determination of joint rotations.

Characterization tests were performed with a healthy subject while recording the motion of the joint with an optical tracking system (OptiTrack Flex 3, NaturalPoint, Inc. DBA OptiTrack, Corvallis, OR, USA)—equipped with four cameras (see Figure 6)—and the sensor signal, simultaneously. The optical tracking system is able to track the pose of a rigid body in 3D. In particular, it is possible to correlate the position of a moving joint (represented by a *x*-*y*-*z* reference system) with respect to the fixed reference system (*x*_0_-*y*_0_-*z*_0_) of the Optitrack. Given the 3D rotation of two body segments expressed with a quaternion, we can calculate the relative rotation as follows:

(6) $$q_{rel}=q_{1}\cdot q_{2}^{*}$$

Being $q_{rel}$ the relative rotation between body segments, $q_{1}$ the rotation of the first body segment, and $q_{2}^{*}$ the conjugate of the rotation of the second body segment.

Markers were placed on the participants in accordance to the Vicon Plug-In Gait marker setup (Vicon Motion Systems Ltd., Oxford Metrics, Oxford, UK). Once the markers were placed, subsets were used to define the 3D pose of rigid bodies of interest, i.e., thigh, shank, upper foot and lower foot. Indeed, the output of the system was a quaternion for each rigid body, which allowed analyzing the relative rotations of single segments.

In order to analyze the ankle rotation, we converted its quaternion to Euler angles, as described in Annex A. In the knee case, we considered only one angle since we focused on the predominant rotation (i.e., the one that is described in the sagittal plane). These considerations are a direct consequence of modelling the knee as a joint having a single Degree of Freedom (DoF). On the other hand, the ankle can be modeled as a 3 DoFs joint. These assumptions are enough for the purpose of lower limb motion estimation. As an example, in order to extract the knee joint angle, the relative quaternion can be transformed to angle/axis representation in the following way:

(7) $$\theta_{knee}=2\cdot \arctan\left(\frac{\sqrt{q_{i}^{2}+q_{j}^{2}+q_{k}^{2}}}{q_{r}}\right)$$

Being $\theta_{knee}$ the knee bending flexion angle, and $q_{i},\;q_{j},\;q_{k},\;q_{r}$ the components of the relative rotation quaternion calculated with Equation (7).

Upon comparing the capacitive sensor measurements with those acquired by the optical tracking system, some data pre-processing was performed. Indeed, the OptiTrack sampling frequency was 65 Hz, while the capacitive sensor signals were produced at 10 Hz.

Using the previous angles and the sensor readings, the behavior of the sensors was approximated by using a polynomial equation. The coefficients were calculated using the least square method. Moreover, for comparison, the polynomial equations using 3rd, 4th and 5th degrees were calculated. The aim of these tests is to demonstrate the validity of the proposed approach, and to show that by using a proper fitting for each DoF, these modules can be then used in real applications, as described in Section 3.5 and Section 3.6.

More details on the developed and applied algorithms (in particular for the 3 DoFs joint) are described in Annex A.

### 3.3. Knee Module Characterization

**Figure 7:**
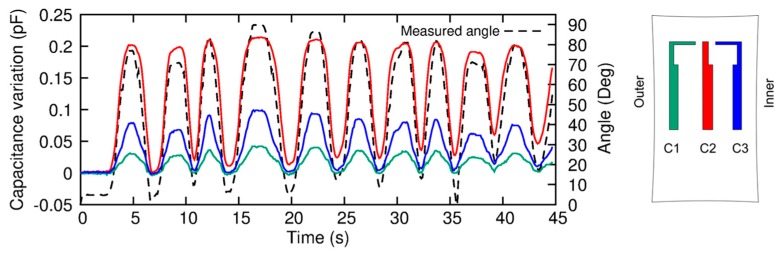
Capacitance variations of the three sensors (green, red and blue solid lines for C1, C2 and C3, respectively) compared with the angle (black dashed line) measured by the tracking optical system.

The sensorized knee brace was worn by one healthy subject (see Figure 7) who performed five cycles of knee flexion/extension according to the following experimental procedure:

1. The participant was asked to extend the knee;
2. The strain sensors and the optical tracking system were reset to remove the offset;
3. The subject performed 10 flexion/extension movements, and data from sensors and tracking system were recorded;
4. The participant stopped the movement, and data recording was stopped.

In Figure 7 the results are shown; in particular, the capacitance variations of the three strain sensors (see the color legend in Figure 7D) are compared with the measured angle (black dashed line). The correlation between the sensor outputs and the bending angle is well visible, with the C2 signal amplitude higher with respect to the C1–C3 responses. This can be easily explained by the higher strain induced in the central knee region during the movements performed by the user. This is also the maximum strain observed in all monitored movements. Indeed, from the sensor output, a strain less than 30% can be extracted. 

**Figure 8:**
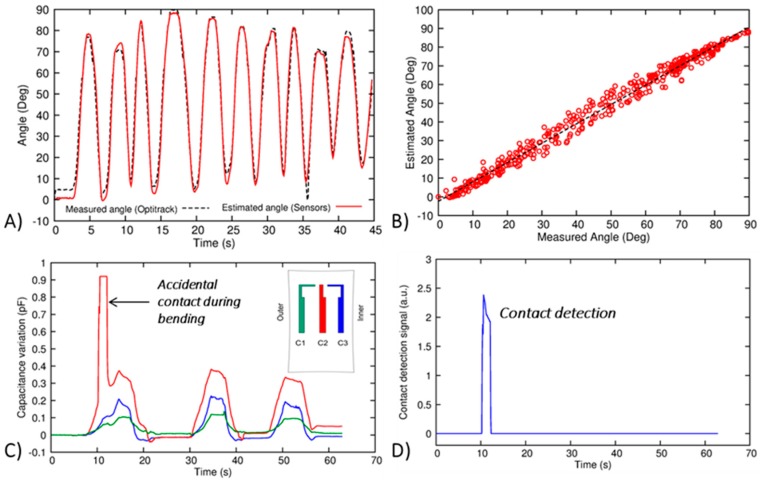
(**A**) Estimated angle (red solid line) obtained by the combination of the three sensors on the kneepad, compared with the angle measured by the OptiTrack system (black dashed line) during bending/unbending cycles; (**B**) Combined output characteristics of the sensorized kneepad; (**C**) Accidental contact during bending, occurring on C2 (red solid line); (**D**) Contact detection signal, reconstructed by comparing the amplitudes of single sensor outputs.

The sensor redundancy can be exploited in several ways. First of all, it can be used for obtaining a higher correlation with the bending angle. Combining the sensor signals a correlation in the full 0–90° range with a root mean square error (RMSE) less than 4° is obtained. In Figure 7A,B the time response and the output characteristics are shown, respectively. In this case, a first order polynomial fitting (i.e., a linear combination) is sufficient to obtain such results. In addition, the redundancy is useful to detect accidental physical contacts on the knee. For instance, in Figure 8C the responses of the three sensors during bending/unbending cycles are shown, where the central sensor C2 is (e.g., contacted by an external object (e.g., accidental obstacle). The sensor output has an abrupt peak due to the pressure caused by the contact. Hence, the signal amplitudes are compared (since we know the correlation between each single sensor with the bending angle), and the occurrence of such contact is detected. In case of pure bending, the difference between the output amplitudes of each couple of sensors is always below a specific threshold that is determined during an initial calibration phase. For instance, in our experiments, a threshold around 0.2 pF has been observed. A tolerance factor (i.e., between 2 and 3) is used so that—if one of the abovementioned differences exceeds the threshold—the system is enabled to detect external contacts. This algorithm can be easily implemented in real time in the microcontroller, with a very low computational effort. As shown, in Figure 8C, the amplitude of C1 raises up to 1 pF, while the remaining signals are well below 0.2 pF. The output of the contact detection signal is shown in Figure 8D. This feature can be used for sending alerts to a potential exoskeleton system [42] in order to monitor, and possibly correct or avoid, movements that could be harmful to the user. 

### 3.4. Ankle Module Characterization

An experimental protocol, similar to the one used for testing the kneepad, was adopted to test the sensorized ankle brace (see Figure 4), as follows:

1. The subject was asked to put the foot on the floor trying to keep a normal angle with the lower leg;
2. The sensors and the tracking system were reset to remove the offset;
3. The participant performed dorsi/plantar flexion, abduction/adduction, rotation or mixed movements (each movement was repeated 10 times);
4. The subject interrupted the movement and data recording was stopped.

As previously anticipated, up to three degrees of freedom are involved in the foot movements, and for this reason, a higher sensor redundancy is needed, in order to correlate the output signals with the Euler angles. Figure 6 shows how the original reference system was oriented with respect to the ankle. Again, more details on the applied rotations can be found in the Supplementary Information.

**Figure 9:**
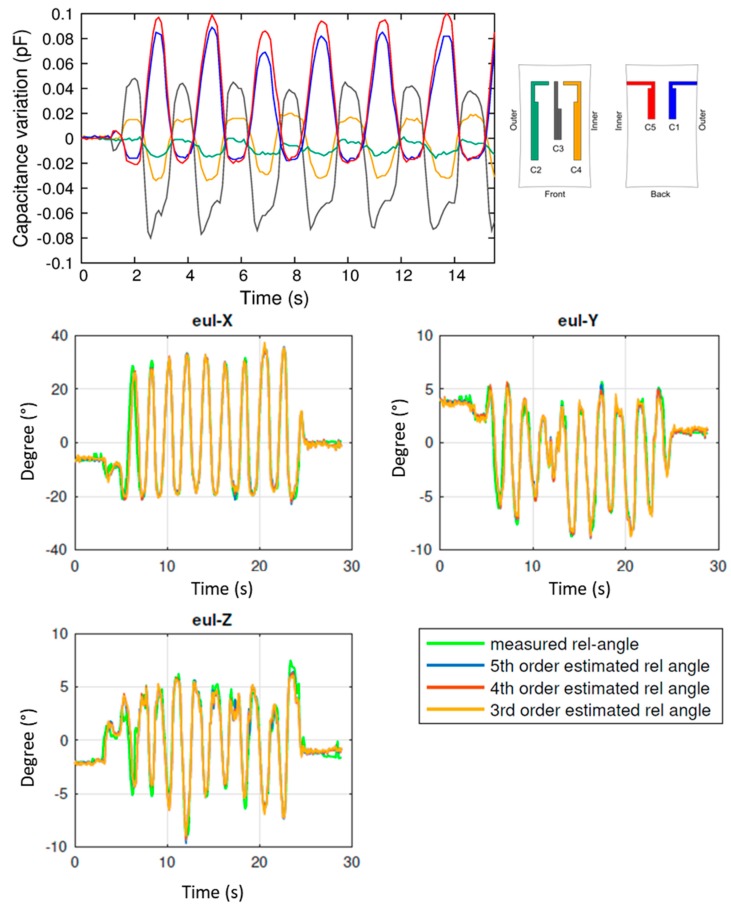
Results of the experimental analysis performed with the anklet during plantar dorsi/flexion. **Top panel**: raw capacitance variations of sensors C1 to C5. **Bottom panels**: reconstruction of the Euler angles (Eul-X, Eul-Y, Eul-Z), and comparison with the values (in green) measured by the optical tracking system.

First of all, the dorsi/plantar flexion was characterized. This movement has one DoF (the rotation γ about the *x* axis, according to the convention, also indicated as Eul-X in Figure 6) that varies much largely with respect to the other two angles. Results are reported in Figure 9. In particular, in the top panel raw capacitive data are shown (see color legend on the right for the correspondent sensor location). Then, the five sensor outputs are combined, using a 3rd, 4th, and 5th order polynomial for fitting the measured angle. Results are reported in the bottom panels of Figure 9. In all cases, the correlation is very high, with a RMSE lower than 3° for Eul-X, and less than 1° for Eul-Y and Eul-Z, this resulting in less than 5% for *x*, and 7% in the other cases.

**Figure 10:**
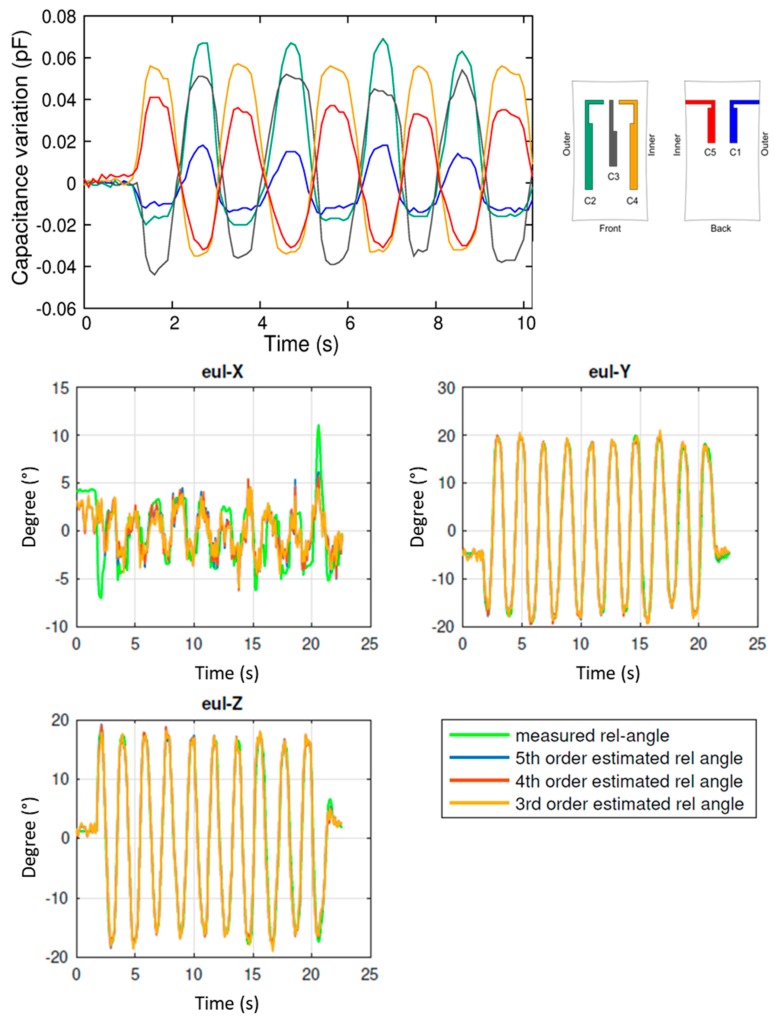
Results of the experimental analysis during plantar abduction/adduction during. **Top panel**: raw capacitance variations of sensors C1 to C5. **Bottom panels**: reconstruction of the Euler angles (Eul-X, Eul-Y, Eul-Z), and comparison with the values (in green) measured by the optical tracking system.

The same procedure was repeated for abduction/adduction movement. Results are shown in Figure 10 and, in this case, Eul-X has a lower variation, as expected. The same polynomial fitting strategy is adopted, giving reliable results also in this case, with a RMSE less than 2° for the all angles. 

**Figure 11:**
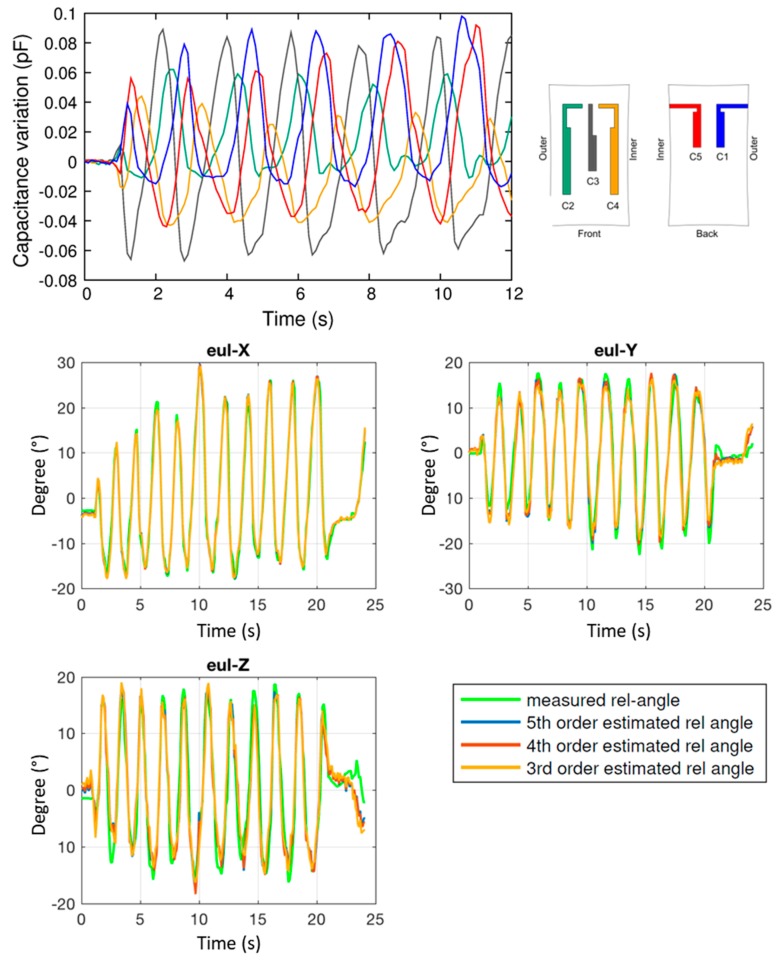
Results of the experimental analysis performed with the anklet during a rotation movement. **Top panel**: raw capacitance variations of sensors C1 to C5. **Bottom panels**: reconstruction of the Euler angles (Eul-X, Eul-Y, Eul-Z), and comparison with the values (in green) measured by the optical tracking system.

Analogously, complete foot rotation is characterized, with the results reported in Figure 11. In this case, as expected, the amplitude of all three angles is in the same range, and the polynomial fitting give results with RMSE lower than 3° for all angles.

**Figure 12:**
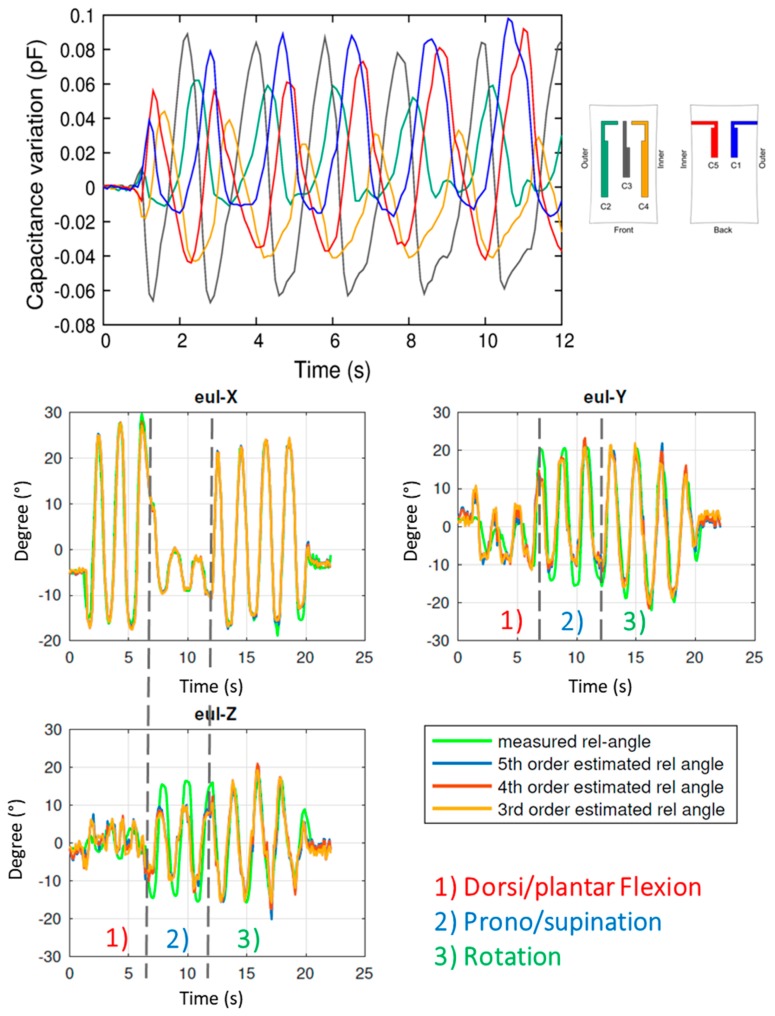
Mixed movement monitoring. **Top panel**: Raw capacitance variations. **Bottom panels**: Reconstructed Euler angles compared with the optical tracking system measurements (solid green line). In this case, different movements can be distinguished.

Finally, tests with the three previous movements combined together were performed. The aim was to verify whether the reconstruction algorithm, together with the three angle estimation, was able to distinguish between these tasks. Results are shown in Figure 12. Three different phases are clearly visible from the angle graphs. In particular, in phase 1, which corresponds to dorsi/plantar flexion, Eul-X variation is much larger with respect to the variation of the other two components. Otherwise, in phase 2, Eul-X has the lowest variations. This behavior corresponds to an abduction/adduction movement, as shown previously. Then, in phase 3 all angles vary in the same range, indicating that a foot rotation is occurring.

**Figure 13:**
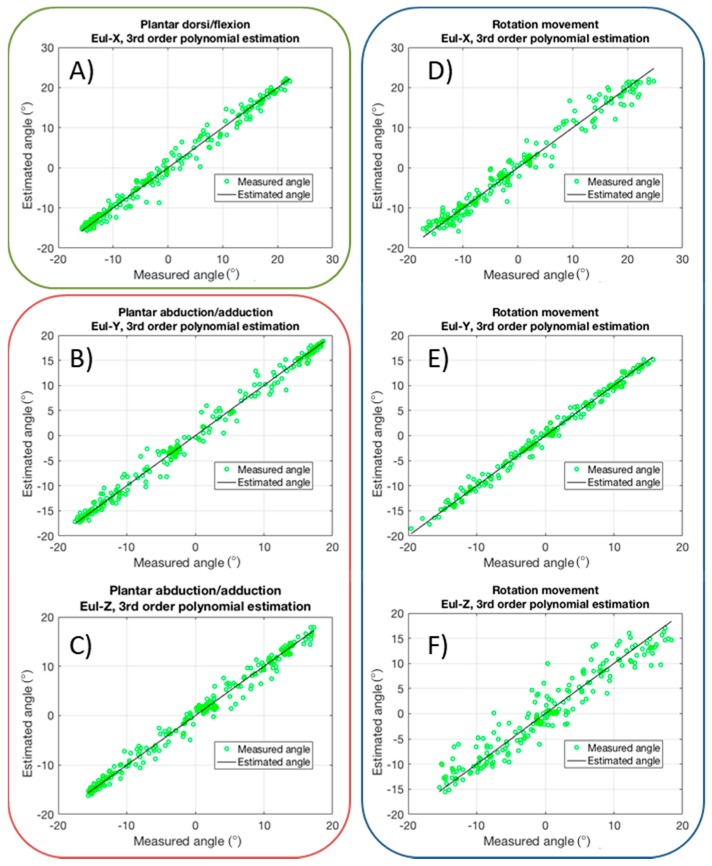
Output characteristics for main angles in different movements using third order polynomial fitting estimation: Eul-X (**A**) for dorsi/plantar flexion; Eul-Y (**B**) and Eul-Z (**C**) for abduction/adduction; Eul-X (**D**), Eul-Y (**E**), Eul-Z (**F**) for rotation.

**Figure 14:**
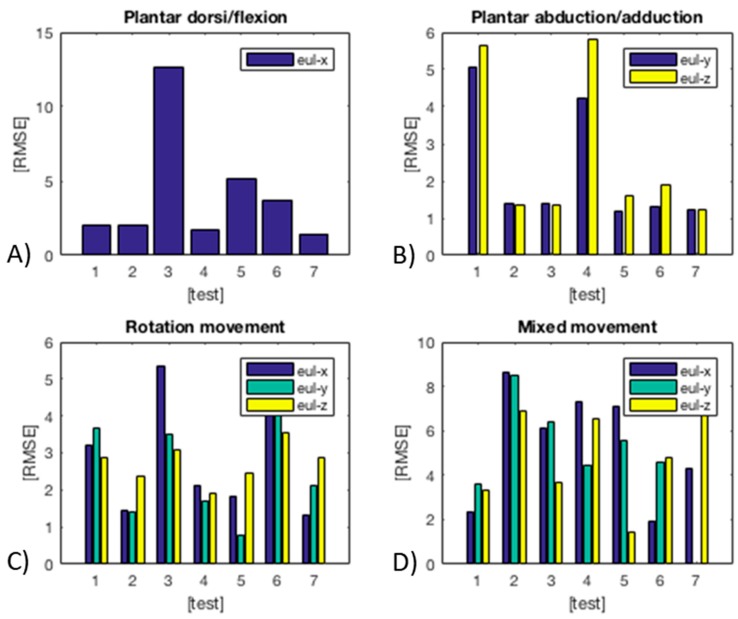
RMSE in seven different tests for each movement: (**A**) Plantar dorsi/flexion; (**B**) Plantar abduction/adduction; (**C**) Rotation; (**D**) Mixed movements.

In Figure 13, the characteristics of the main angles for each of the tested movements are shown. In particular: Eul-X for dorsi/plantar flexion; Eul-Y and Eul-Z for abduction/adduction; and, Eul-X, Eul-Y and Eul-Z for rotation. In all cases, a third order polynomial fitting was used, showing that this choice gives good results and a low RMSE. Moreover, Figure 14 shows the RMSE in seven different tests for each kind of movement. The modules have been tested with two healthy subjects, then these data cannot offer statistical meaning at this stage. However, the resulting low RMSE for all the tests demonstrates the potentiality of this method for monitoring human movements. 

### 3.5. Knee Activity Monitoring

**Figure 15:**
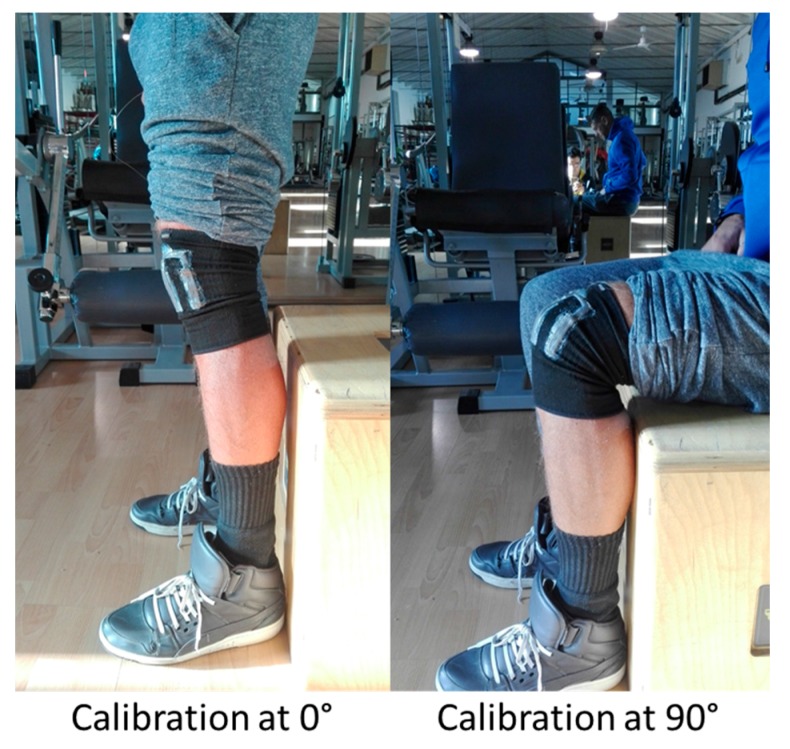
Kneepad calibration.

As demonstrated in Section 3.3, the knee module can monitor the bending flexion angle with a good accuracy. First of all, the knee module can be calibrated with the following procedure:

1. After wearing the kneepad, the electronic board is connected to the module and the acquisition system is switched on;
2. The subject stands up, keeping the knee completely extended, as shown in Figure 15. Thus, the sensor outputs are offset to 0°;
3. The subject sits down, keeping the knee flexed at 90°, using a seat having legs perpendicular to its plane, as shown in Figure 15. Then, the output values for 90° are recorded by the system.

As demonstrated in Section 3.2, the output characteristic is linear (see Figure 8B), then all intermediate angles can be acquired by using a linear fitting algorithm. This approach is easily repeatable whenever the module is worn, and does not require any external instrumentation for its calibration. Moreover, to monitor properly faster movements, the sensing acquisition rate has been increased to 50 Hz.

Then, to investigate its potential exploitation in long-term motion monitoring, as a preliminary study we tested some specific tasks, typical of daily and sport activities. In particular, walking, running on a treadmill, leg extension, and squat. All these tasks have been performed by a user different from the one wearing the smart garments validated at Optitrack (see Section 3.3 and Section 3.4).

**Figure 16:**
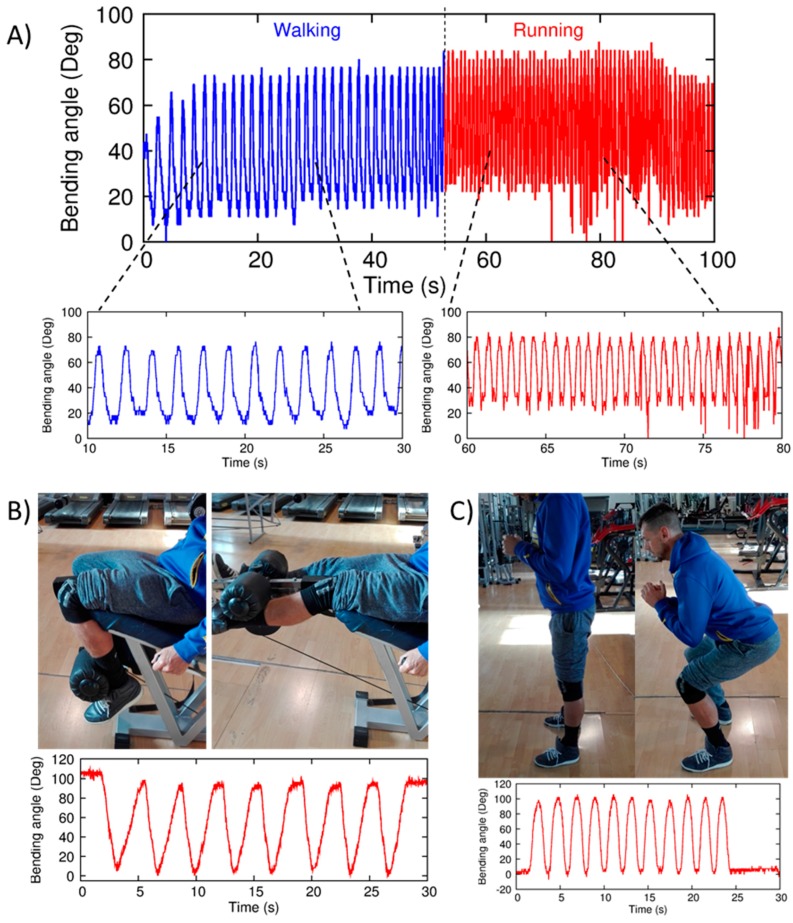
Knee monitoring during different tasks: (**A**) Walking/running on a treadmill; (**B**) Leg-extension exercises; (**C**) Squat exercises.

Kneepad responses for walking and running cycles are shown in Figure 16A. Both movements are detected correctly, also in the case of running cycles, due to the increased acquisition rate. Then, in Figure 16B,C, output signals due to leg-extension and squat exercises are shown. In both cases, the whole movement range is detected.

### 3.6. Ankle Activity Monitoring

**Figure 17:**
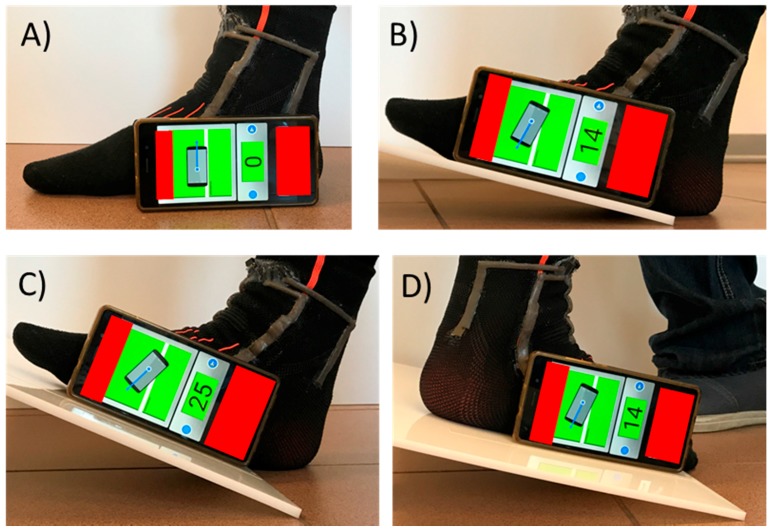
Example of possible calibration procedure for anklet using a smartphone and a customized application.

As demonstrated in Section 3.4, polynomial fitting can be used for retrieving ankle DoFs. However, the fitting parameters can vary largely in different subjects and after each wearing. Then, in this section, a potential calibration procedure is proposed that does not require an optical measurement system, and that can be performed directly by the user after wearing the module. In particular:

1. The anklet is worn and, after the connection to the electronic acquisition system, all sensors output are offset with the user standing up, as shown in Figure 15A. This is considered the 0° value for all DoFs;
2. The user acquires the output signals for some angles that can be measured using a smartphone and a proper application. Some examples are shown in Figure 17B–D;
3. The acquired outputs can be used by the measuring system to fit calibration parameters. Then, the full angle range of each DoF can be measured by the system.

In future work, a customized smartphone application can be developed. It should guide the user on the different positions of the foot needed for the calibration, acquire sensor outputs and then implement the proper polynomial fitting for measuring the full ranges. In Figure 15, a potential calibration procedure for the angle parallel to the sagittal plane, using an already available application is shown. In particular, by acquiring the output signals of four different angles (i.e., 0°, +14°, +25° and −14°), a third polynomial fitting can then be performed.

This procedure can compensate the anatomical characteristics of users, the different positioning of the anklet after each donning, and the variability of single sensor characteristics (i.e., dielectric thickness, positioning of single sensors during the fabrication process).

## 4. Discussion and Conclusions

In this work we designed and developed two sensorized soft modules for monitoring lower limb movements. In particular, the knee brace, embedding three capacitive strain sensors, is able to detect the knee bending with a high accuracy (RMSE less than 4°), and to discriminate this from pressures arising by accidental external contacts, by implementing in real time a low computational algorithm. In the next phases of investigation, the authors will address the possibility of using more sensors (i.e., five) at the knee, in order to detect also internal/external rotation and its abduction/adduction angles. This would be important to achieve a complete analysis of knee biomechanics for several healthcare applications. Subsequently the impact of this extended modeling of the knee will be evaluated.

Moreover, the sensorized anklet has a distribution of five soft sensors at both front and back sides for retrieving information about dorsi/plantar flexion, adduction/abduction and rotation. We demonstrate how exploiting the sensor redundancy and polynomial combinations (i.e., 3rd, 4th, and 5th degree were successfully investigated) for reconstructing the three DoFs, all the above-mentioned movements can be monitored and distinguished with a low RMSE (less than 4° in the worst case). From a qualitative evaluation of sensor hysteresis we could observe that it is negligible, however this aspect will be addressed more in detail in the near future.

The soft modules show an excellent wearability, since the distributed soft sensing at the joints relies on a fabrication process based on commercial sport braces and the integrated sensors do not affect their stretchability. The commercial braces are designed to adhere to the body in a comfortable way, and negligible sliding after activities was observed. In any case, the calibration procedure can be easily repeated after some time if the user moves the braces or a remarkable sliding occurs. The anklet adhesion is very reliable, since also the foot anatomy limits sliding of the brace.

Furthermore, the electronics for each module is removable and this facilitates the donning and doffing procedure. The connections to read-out electronics are made using the same conductive textile of sensing elements. In this way, from the mechanical point of view, the robustness is higher, since the rigid parts are minimized with respect to using typical coaxial wires, but also because sensors and wiring result from the same fabrication process on the garment. From the electrical point of view, the parasitic capacitances are kept as low as possible by minimizing the wiring length. In this work, however, we do not address the washability of the modules. Silicon rubbers were used, and although this material alone can be compatible with a washing process, the latter could affect the adhesion of the sensors to the textile and this issue would need a deeper investigation from a material science point of view. On the other hand, in the near future our purpose is to extend the capabilities of the textile parts of the sensing system as much as possible. Indeed, the conductive parts of the transducers are built with textiles, and along this line, the fabric conductive electrodes will be directly sued in the garment in a way to isolate them from the user and outside world (while dielectric and passivating materials robust to washing cycles will be employed).

As regards the robustness of the devices, since the sensors’ outputs consist of relative capacitance variations, any slight variations of absolute capacitance values (due to e.g., manual alignment, deposition of uncured Ecoflex^®^ with low control on the thickness, mismatch on the sensor positioning, variability due to different people size) do not affect the sensing function nor reliability. With the current fabrication process, after several tests, partial detachment of sensors was observed (especially at the corners). This is due to the stress induced by the movements in the lower Ecoflex^®^ layer. In the future, also this aspect will be improved by exploiting textile engineering techniques as abovementioned. Additionally, in the second stage of this work, the sampling rate has been increased to 50 Hz. As shown in Section 3.5, this is needed for monitoring fast movements such as running cycles.

More than 60 tests were performed recording both the sensor measurements and the optical ones. In particular, for the ankle the polynomial fitting gave satisfactory results in more than 90% of the tests. The tests were performed using two healthy subjects; hence at this stage results do not offer statistical meaning. Indeed, this work is a study for the design and characterization of the sensors, with the goal of demonstrating the capability of the smart garments to replicate the optical tracking measurements. In this work, we demonstrated that a polynomial fitting can be used for retrieving angles at lower limb joints. Then, in order to determine the fitting parameters, as described in Section 3.5 and Section 3.6, we are currently working on a calibration procedure that does not require the use of optical motion capture. It can be performed by the user himself/herself after donning the module, by using a smartphone and a customized application.

Starting from the encouraging results obtained so far our research will be directed towards gathering statistical data across multiple users. The gathered data will serve as input for the optimization of the layout of both modules. Moreover, more complex tasks (i.e., fast walking, negotiating stairs, sitting/standing will be studied and characterized. In particular, the two modules will be used simultaneously in order to provide biomechanical information about the whole lower limb movement. First, the smart garments will be employed for providing sensory feedback in a lower limb exoskeleton that will be developed to provide assistance to users with mobility impairments. This is actually being addressed within the EU XoSoft project [42,43,44] by means of user centered design [46] with ICT approaches enabling an easy and comfortable to wear garment for promoting quality of life. Still in the rehabilitation field, the devices could be used as individual modules for providing feedback about abnormal posture or movement due to specific pathologies and injuries in the different anatomical planes (e.g., anklet). Moreover our results could be applied for human movement analysis in sport to monitor and improve athletes’ performance, as well as to track user movements in gaming.

## Appendix A

### Appendix A.1. Reconstruction Algorithm

In the following, the reconstruction algorithm for reconstructing the joint angles is described. In particular, for a given time, sensor outputs were combined in order represent the angles measured by the OptiTrack. The transformation is given by Equation (A1), in which matrix A represents the sensor output signals, vector x is the estimated equation coefficient, and vector b are the OptiTrack outputs in quaternions:

(A1) Ax=b

By means of the pseudo-inverse of matrix A ($A^{+}$), it is possible to find the best fit (least squares) solution to a system of linear equations as shown in Equation (A2). Therefore, the equation coefficients can be estimated with *A^+^*.

(A2) $$x=A^{+}b$$

Different equations are characterized by different types of A and x. As an example, in case of a 1st polynomial equation, the system described by Equation (A2) becomes:

(A3) $$\left[\begin{array}{c} C_{3}\left(t_{1}\right)\\ C_{3}\left(t_{2}\right)\\ \begin{array}{c} C_{3}\left(t_{3}\right)\\ \begin{array}{c} \vdots \\ C_{3}\left(t_{N}\right) \end{array} \end{array} \end{array}\begin{array}{c} 1\\ 1\\ \begin{array}{c} 1\\ \begin{array}{c} \vdots \\ 1 \end{array} \end{array} \end{array}\right]\left[\begin{array}{c} m\\ n \end{array}\right]=\left[\begin{array}{c} angle\left(t_{1}\right)\\ angle\left(t_{2}\right)\\ \begin{array}{c} angle\left(t_{3}\right)\\ \vdots \\ angle\left(t_{N}\right) \end{array} \end{array}\right]$$

where $C_{3}\left(t_{1}\right)$ represents the signal of sensor $C_{3}$ at time $t_{1}$, $angle\left(t_{1}\right)$ represents the angle at the same time, thus the resulting estimated equation is:

(A4) $$angle\left(t\right)=mC_{3}\left(t\right)+n$$

Otherwise, if a second order polynomial is considered, Equation (A2) becomes:

(A5) $$\left[\begin{array}{c} C_{3}^{2}\left(t_{1}\right)\\ C_{3}^{2}\left(t_{2}\right)\\ \begin{array}{c} C_{3}^{2}\left(t_{3}\right)\\ \begin{array}{c} \vdots \\ C_{3}^{2}\left(t_{N}\right) \end{array} \end{array} \end{array}\begin{array}{c} C_{3}\left(t_{1}\right)\\ C_{3}\left(t_{2}\right)\\ \begin{array}{c} C_{3}\left(t_{3}\right)\\ \begin{array}{c} \vdots \\ C_{3}\left(t_{N}\right) \end{array} \end{array} \end{array}\begin{array}{c} 1\\ 1\\ \begin{array}{c} 1\\ \begin{array}{c} \vdots \\ 1 \end{array} \end{array} \end{array}\right]\left[\begin{array}{c} m\\ n\\ k \end{array}\right]=\left[\begin{array}{c} angle\left(t_{1}\right)\\ angle\left(t_{2}\right)\\ \begin{array}{c} angle\left(t_{3}\right)\\ \vdots \\ angle\left(t_{N}\right) \end{array} \end{array}\right]$$

It is straightforward that it is possible to estimate polynomial equations having a proper order so to fit the measured data. Finally, by linking different sensor measurements and different equations coefficients (i.e., *A^+^* and *b* in Equation (A2), respectively) it is possible to combine different sensors to better estimate the angle, as shown in Equation (A6):

(A6) $$\left[\begin{array}{ccccc} C_{3}\left(t_{1}\right) & 1 & & C_{4}\left(t_{1}\right) & 1\\ C_{3}\left(t_{2}\right) & 1 & & C_{4}\left(t_{2}\right) & 1\\ C_{3}\left(t_{3}\right) & 1 & & C_{4}\left(t_{3}\right) & 1\\ \vdots & \vdots & & \vdots & \vdots \\ C_{3}\left(t_{N}\right) & 1 & & C_{4}\left(t_{N}\right) & 1 \end{array}\right]\left[\begin{array}{c} m_{3}\\ n_{3}\\ \begin{array}{c} m_{4}\\ n_{4} \end{array} \end{array}\right]=\left[\begin{array}{c} angle\left(t_{1}\right)\\ angle\left(t_{2}\right)\\ \begin{array}{c} angle\left(t_{3}\right)\\ \vdots \\ angle\left(t_{N}\right) \end{array} \end{array}\right]$$

### Appendix A.2. From Quaternion to Euler Angles

Euler rotations are used to split complete 3D rotations of the coordinate system into three elementary rotations. The latter may be described according to different conventions depending on the axes about which the rotations are carried out and their sequence. In this work the ZYX intrinsic convention. The term intrinsic denotes rotations that occur about the axes of a coordinate system attached to a moving body. The initial orientation of the coordinate system overlaps the world frame, denoted like *x*_0_-*y*_0_-*z*_0_. Then, according to the ZYX convention, a first rotation α is performed about the *z* axis. The result of this rotation is that the frame of the coordinate system are described by a frame denoted like *x*_1_-*y*_1_-*z*_1_ where *z*_1_ = *z*_0_. A second rotation β takes place around the new *y* axis (*y*_1_) and, finally, γ occurs about the new *x* axis (*x*_2_). Therefore, the sequential application of the α, β and γ rotations result in the description of the orientation of a rigid body in 3D space with respect to the World frame. The rotation matrices that describe the abovementioned rotations are reported in the following table:

(A7) $$R_{z}\left(\alpha \right)=\left[\begin{array}{ccc} \cos\alpha & \sin\alpha & 0\\ -\sin\alpha & \cos\alpha & 0\\ 0 & 0 & 1 \end{array}\right]$$

(A8) $$R_{y}\left(\beta \right)=\left[\begin{array}{ccc} \cos\beta & 0 & -\sin\beta \\ 0 & 1 & 0\\ \sin\beta & 0 & \cos\beta \end{array}\right]$$

(A9) $$R_{x}\left(\gamma \right)=\left[\begin{array}{ccc} 0 & 0 & 0\\ 0 & \cos\gamma & \sin\gamma \\ 1 & -\sin\gamma & \cos\gamma \end{array}\right]$$

Finally, in the following the equations for calculating Euler angles from quaternion components:

(A10) $$\alpha =atan\left(\frac{2\left(q_{r}q_{k}+q_{i}q_{j}\right)}{1-2\left(q_{j}^{2}+q_{k}^{2}\right)}\right)$$

(A11) $$\beta =asin\left(2\left(q_{r}q_{j}-q_{i}q_{k}\right)\right)$$

(A12) $$\gamma =atan\left(\frac{2\left(q_{r}q_{i}+q_{j}q_{k}\right)}{1-2\left(q_{i}^{2}+q_{j}^{2}\right)}\right)$$
